# How and how much does RAD-seq bias genetic diversity estimates?

**DOI:** 10.1186/s12862-016-0791-0

**Published:** 2016-11-08

**Authors:** Marie Cariou, Laurent Duret, Sylvain Charlat

**Affiliations:** 1 Université de Lyon, Université Lyon 1, CNRS, UMR 5558, Laboratoire de Biométrie et Biologie Evolutive, 43 boulevard du 11 novembre 1918, Villeurbanne, F-69622 France; 2 Current address: Laboratory of Evolutionary Genetics and Ecology, URBE, University of Namur, Rue de Bruxelles 61, 5000 Namur, Belgium

**Keywords:** Population genomics, Reduced representation genomics, Allele drop-out, ABC, Non-neutral model, Population structure

## Abstract

**Background:**

RAD-seq is a powerful tool, increasingly used in population genomics. However, earlier studies have raised red flags regarding possible biases associated with this technique. In particular, polymorphism on restriction sites results in preferential sampling of closely related haplotypes, so that RAD data tends to underestimate genetic diversity.

**Results:**

Here we (1) clarify the theoretical basis of this bias, highlighting the potential confounding effects of population structure and selection, (2) confront predictions to real data from *in silico* digestion of full genomes and (3) provide a proof of concept toward an ABC-based correction of the RAD-seq bias. Under a neutral and panmictic model, we confirm the previously established relationship between the true polymorphism and its RAD-based estimation, showing a more pronounced bias when polymorphism is high. Using more elaborate models, we show that selection, resulting in heterogeneous levels of polymorphism along the genome, exacerbates the bias and leads to a more pronounced underestimation. On the contrary, spatial genetic structure tends to reduce the bias. We confront the neutral and panmictic model to “ideal” empirical data (*in silico* RAD-sequencing) using full genomes from natural populations of the fruit fly *Drosophila melanogaster* and the fungus *Shizophyllum commune*, harbouring respectively moderate and high genetic diversity. In *D. melanogaster*, predictions fit the model, but the small difference between the true and RAD polymorphism makes this comparison insensitive to deviations from the model. In the highly polymorphic fungus, the model captures a large part of the bias but makes inaccurate predictions. Accordingly, ABC corrections based on this model improve the estimations, albeit with some imprecisions.

**Conclusion:**

The RAD-seq underestimation of genetic diversity associated with polymorphism in restriction sites becomes more pronounced when polymorphism is high. In practice, this means that in many systems where polymorphism does not exceed 2 %, the bias is of minor importance in the face of other sources of uncertainty, such as heterogeneous bases composition or technical artefacts. The neutral panmictic model provides a practical mean to correct the bias through ABC, albeit with some imprecisions. More elaborate ABC methods might integrate additional parameters, such as population structure and selection, but their opposite effects could hinder accurate corrections.

**Electronic supplementary material:**

The online version of this article (doi:10.1186/s12862-016-0791-0) contains supplementary material, which is available to authorized users.

## Background

Reduced representation genomics aim at sequencing particular parts of the genomes of many individuals, rather than full genomes of one or a few individuals, in a single sequencing reaction. One such approach, RAD-seq (and related protocols) makes use of restriction enzymes to target DNA regions flanking cut sites that are more or less randomly distributed throughout the genome [1, 2]. Among other applications, this technique can provide genome wide estimates of population genetic diversity. Previous studies, however, have emphasized that RAD-seq diversity estimates can be systematically biased [3–5], impeding the use of RAD-seq as a standardised tool to measure and compare genetic diversity across study systems. First, heterogeneity in base composition along genomes implies that any particular cut site deviates to some extent from a random distribution across the genome [6]. Because base composition and polymorphism can themselves be linked (e.g. lower GC content in neutral and thus more polymorphic regions), this can impact diversity estimates. Particular motifs present in the restriction site might also be enriched in some particular regions of the genomes (e.g. motifs corresponding to protein domains [7]).

Arguably, such biases probably exist for any kind of molecular marker, because of the inherent contradiction between “targeted” and “random” sequencing. But RAD-seq also presents an additional bias caused by polymorphism on restriction sites; just as its ancestor, the AFLP technique, although in a more subtle way [8, 9]. With the AFLP, any loss of restriction site turned an heterozygous to a seemingly homozygous genotype. In RAD-seq, sequencing depth can be used to identify such cases, and the presence/absence of restriction sites is not the primary source of information. Nevertheless, this Allele Drop Out (ADO) leads to underestimate the polymorphism, because of the linkage disequilibrium between the restriction sites and SNPs within the RAD sequences. In more simple terms, individuals or haplotypes that are more closely related than the population average tend to share the same state at the restriction site (presence or absence), and are thus over-represented in RAD-seq datasets.

Here we focus on this latter bias, hereafter simply referred to as “the RAD-seq bias”. The impact of ADO has been investigated in several earlier studies [10, 11], where difference in coverage between loci was proposed as a solution to detect null alleles. Here we do not consider this option, which requires a high sequencing depth that is not always achieved. We rather aim at a better understanding of this bias, through the confrontation of simulated and empirical data. Simulations were first performed under a Wright-Fisher neutral and panmictic model, in order to confirm the previously established relationship between the true polymorphism and its RAD-based estimate. We further explored the potential consequences of deviations from a neutral and panmictic model. We show that population structure tends to reduce the RAD-seq bias, because RAD-seq underestimates divergence *within* but not *between* populations. In contrast, variations in polymorphism along the genome, which is a typical signature of selective constraints, tend to intensify the RAD-seq bias, because regions of low polymorphism contribute disproportionally to the data.

We then confronted theoretical predictions to ideal empirical data, that is, *in silico* digestions of full genomes from natural populations of the fruit fly *Drosophila melanogaster* (DPGP [12]), harbouring moderate polymorphism, and the fungus *Schizophyllum commune* [13], harbouring high polymorphism. These two case studies generally confirm the expected relation between the level of polymorphism and the intensity of the RAD-seq bias: the bias is much stronger in the highly polymorphic species. In *D. melanogaster*, the bias is not intense enough to assess potential deviations from the neutral and panmictic model. In *S. commune* this model captures a large part of the bias, but the observed RAD polymorphism falls out of its predicted distribution. Accordingly, ABC corrections based on this model are satisfactory in *D. melanogaster*, but less accurate in *S. commune*. Although our results confirm those of previous studies having raised red flags regarding the RAD-seq bias [8, 9], we would argue that in many species, where polymorphism is low, this problem is of negligible importance in the face of other sources of uncertainty. In very polymorphic species, our ABC correction can mitigate the bias, although population structure, selection, or yet unidentified additional factors, introduce some imprecision in this correction.

## Methods

### Simulations and genetic diversity measures

To measure the theoretical impact of the RAD-seq bias, we simulated sequence data and retrieved RAD tags *in silico*. Each simulation consisted in the generation of coalescents for 1000 genetically unlinked loci, with complete linkage within loci, in four haploid lineages, using the *ms* programme [14]. *Seq-gen* was then used to produce sequences of 10 kb for each locus [15]. To generate RAD-seq data, we randomly merged by pairs the four haploid genomes to form two diploids, and searched ten randomly defined restriction sites of 8 bp (searching for more than one motif increases the number of RAD loci without increasing the alignment size and computing time). This yielded an average of 1500 RAD loci of 100 bp per genome.

In the first model, we assumed the population was diploid, unstructured, and θ, the population mutation rate (4*Ne*μ) was homogeneous along the genome. In a second model, we explored the potential consequences of selection by assigning different θ values to different groups of loci. Specifically, we assumed that 70 % of the genome had a given θ value, while θ was twice smaller in 20 % of the genome, and 10 times smaller in the remaining 10 % of the genome. To mimic variations in the fraction of coding regions and selection intensity, similar simulations were run with other proportions (50, 40 and 10 % instead of 70, 20 and 10 %) and even more heterogeneous θ (reduced 10 fold and 100 fold instead of 2 fold and 10 fold). In these simulations, π_true_ is the mean of the θ values, weighted by their respective proportions in the genome. Finally, in a third model, we assumed θ was homogeneous along the genome but introduced spatial structure by sampling the two diploid genomes in two populations having diverged for a time *t*. For all simulations, θ values were randomly sampled from a uniform distribution between −5 and −1 of log10(θ), thus corresponding to θ values ranging from 10^−5^ to 10^−1^ (program commands are provided in supplementary materials).

To measure the RAD-seq bias in real data, we performed *in silico* RAD-seq experiments, using full genome sequences from natural populations of *Drosophila melanogaster* [12] and *Schizophyllum commune* [13]. For both species, the sequences that are available correspond to haploid genomes. To mimic real RAD-seq experiments, which are generally performed on diploid individuals, we randomly selected pairs of haploid genomes coming from the same population, to generate diploid individuals (Additional file 1: Table S1). RAD tags were then retrieved from each individual.

In all analysis, we calculated π, the nucleotide diversity, as the average genetic distance across loci between two diploid specimens. This distance was either computed from sequences associated with an intact restriction site that would actually be retrieved in a RAD experiment (π_RAD_) or from all sequences at the same loci (π_true_). This later value should thus represent an unbiased measure of nucleotide diversity at RAD loci. We evaluate the intensity of the RAD-seq bias by comparing π_RAD_ with π_true_. Details for these calculations are given below:

$$ \pi ={\displaystyle {\sum}_{k=1}^{k=n}{\pi}_k/{\displaystyle {\sum}_{k=1}^{k=n}{L}_k}} $$

where *n* is the number of loci in that individual, *L*_*k*_ is the length of locus *k* (here *L* = 100 for all RAD loci), and *π*_*k*_ is the genetic distance at locus *k*, calculated as follows:

$$ {\pi}_k=\frac{1}{h1+h2}\ast {\displaystyle {\sum}_{i=1}^{i=h1}{\displaystyle {\sum}_{j=1}^{j=h2}{d}_{ij}}} $$

where *h1* and *h2* are the number of haplotypes present in individuals 1 and 2 (in the case of π_RAD_ calculations) while *h1* = *h2* = 2 for π_TRUE_ calculations. *d*_*ij*_ is the genetic distance between allele *i* and *j*.

In data simulated with spatial structure, the same values (also called π for simplicity) correspond to measures of the divergence between the two subpopulations.

### ABC for the estimation of nucleotidic diversity from RAD data

We used Approximate Bayesian Computations (ABC) to correct RAD-seq estimates of genetic polymorphism. In these simulations, as in our first model, we assumed the population was diploid, unstructured, and θ was homogeneous along the genome. We considered the following summary statistics: (1) π_RADobs_, the observed nucleotidic diversity in RAD-seq data (average distance between individuals) and (2) the proportion of loci in each individual shared with the other one. Calculation of the posterior distribution of θ for each observed dataset was performed with functions from the R package *abc* [16]. We used a tolerance rate of 0.05 and local linear regressions to adjust the accepted simulations to the observed data, and tested our approach by cross-validation.

## Results

### The RAD-seq bias under a neutral and panmictic model

**Fig. 1:**
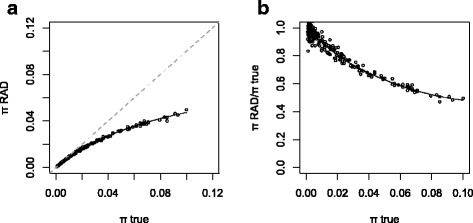
**a** The relation between π_true_, the nucleotidic diversity measured using all individuals at RAD loci, and π_RAD_, measured using only loci associated with an intact restriction site, simulated under a neutral and panmictic model. **b** The relation between the amplitude of the RAD polymorphism bias (π_RAD_ / π_true_) and the level of polymorphism. *Solid lines* represent local linear regressions

In order to validate our approach, we first aimed at retrieving the previously established effects of ADO on the RAD-seq polymorphism in unstructured populations under a neutral model. To this end, RAD-seq data was obtained from simulated genomes. In a panmictic population evolving neutrally, the population mutation rate (θ = 4*Ne*μ) is expected to equal the nucleotidic diversity π, the average distance between haplotypes sampled randomly within the population. In the present analysis, π is measured using pairs of diploid specimens. As expected, we observed in our simulations that π_true_ (the mean genetic distance between individuals at all RAD loci, regardless of the actual state of the restriction site) is an unbiased estimate of θ. However, π_RAD_, measured using sequences flanking intact restriction sites only, is an underestimate of θ; a bias that increases with the level of polymorphism (Fig. 1).

### The RAD-seq bias under non neutral and spatially structured models

Real world populations do not necessarily follow the neutral panmictic model. Some regions of the genome are submitted to more frequent or intense episodes of selection than others, increasing the heterogeneity of the polymorphism along the genome. Population structure can also exist at various degrees, producing more or less pronounced genetic differentiation between sub-populations. In an attempt to provide a more general picture of the plausible scope of the RAD-seq bias, we thus explored the consequences of relaxing the assumptions of neutrality and panmixia.

**Fig. 2:**
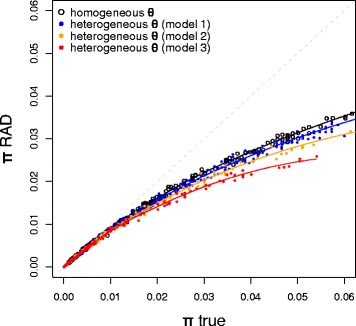
The relation between π_true_ and π_RAD_ in a non neutral model. Black open dots: homogeneous θ values (neutral model, equivalent to Fig. 1). Solid coloured dots: heterogeneous θ values along the genome. Each simulation was run by randomly choosing a reference θ value (θ_ref_), which was used to assign different θ values to different genomic regions, with increasing heterogeneity in the three models. Blue dots: Model 1: 70 % of loci with θ_1_ = θ_ref_, 20 % with θ_2_ = θ_ref_ /2 and 10 % with θ_3_ = θ_ref_ /10; orange dots: Model 2: 70 % of loci with θ_1_ = θ_ref_, 20 % with θ_2_ = θ_ref_ /10 and 10 % with θ_3_ = θ_ref_ /100; red dots: Model 3: 50 % of loci with θ_1_ = θ_ref_, 40 % with θ_2_ = θ_ref_ /10 and 10 % with θ_3_ = θ_ref_ /100. Solid lines represent local linear regressions. The figure shows that the underestimation of genetic diversity is stronger when polymorphism is more heterogeneous along the genome

First, the impact of selection was investigated by imposing heterogeneity in polymorphism along simulated genomes. Using this model, we observe again that π_true_ is an unbiased estimate of θ (even when θ is not homogeneous along the genome) while π_RAD_ is an underestimate. In addition, we find that heterogeneity in θ amplifies the bias (Fig. 2). We propose the following interpretation for this result: in regions with higher polymorphism, the chances of gaining or losing a RAD locus due to mutations at the restriction site are higher, and thus the density of RAD markers shared across individuals in these regions is lower. In other words, genomic regions with a higher degree of polymorphism tend to be under-represented in RAD-seq data.

**Fig. 3:**
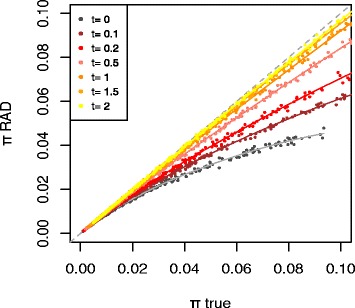
The relation between π_true_ and π_RAD_ in a spatially structured model. Colours indicate divergence time between sub-populations (t), measured in 4*Ne units (that is, the ratio between the divergence time and the average coalescence time). Solid lines represent local linear regressions. The figure shows that the underestimation of genetic diversity is less strong when sub-populations are more divergent

The impact of deviations from panmixia on the RAD-seq bias was investigated using a third model. Here we had the *a priori* intuition that population structure should reduce the intensity of the RAD-seq bias. Indeed, the bias in the sampling of coalescents in RAD-seq data arises from the fact that pairs of haplotypes corresponding to shorter coalescents are more frequently associated with restriction sites having the same state (present or absent), and are thus overrepresented in RAD data. Such a problem should be reduced if RAD-seq is used to estimate divergence among isolated populations, because the age of the population split imposes a lower bound to coalescence time. To take an extreme and illustrative case, the RAD-seq bias should not affect the estimation of genetic divergence among strictly isolated species having evolved separately for far longer than their coalescence time. Simulations confirmed this conjecture (Fig. 3). As expected, the RAD-seq bias (here calculated on the global population) was reduced by spatial structure and the intensity of the bias was inversely related to the time of divergence between sub-populations (here π measures polymorphism within the global population). In brief, RAD-seq underestimates divergence *within* but not *between* populations. This is consistent with the earlier finding of Gautier et al. [8] that ADO leads to overestimate Fst. Indeed, this differentiation index measures the discrepancy between intra and inter-population polymorphism, which means that if only intra-population polymorphism is under-estimated, the bias will increase the Fst.

### Comparing empirical data with simulations

We used *in silico* digestions of full genomes (with phased haplotypes) from natural populations to assess the concordance between simulated and real data. Most species for which such data is available harbour a low to moderate genome-wide diversity (below 2 %), under which circumstances the RAD-seq bias is expected to be negligible. This is for example the case in the fruit fly *D. melanogaster*. In this species, π_true_ ranged from 0.61 % to 0.73 % in the four populations studied. For such values, simulations under the neutral panmictic model predict that π_RAD_ should only be 5 % lower than π_true_ (Fig. 1b). The observed π_RAD_ values fit this prediction, ranging from 0.59 % to 0.70 % in the four populations. However, with so small differences between π_RAD_ and π_true_, this case study provides only limited power to assess the pertinence of the model.

We thus looked for full genome data from natural populations of more polymorphic species, which would provide more informative comparisons between simulated and real data. To our knowledge, the appropriate data exist only for the fungus *Schizophyllum commune* (NB: transcriptome data exist for several other species harbouring high polymorphism [17], but these sequences are not appropriate to mimic a RADseq experiment because i) these datasets do not provide phased haplotypes, and ii) the presence of introns in gene leads to reduce the genetic linkage between sites within mRNAs, which mitigates the RAD-seq bias). Sequences available from this species originate from two populations, from North America and Russia, each characterised by very high polymorphism (π_TRUE_ = 9.7 % and 7.4 %, respectively). Accordingly, π_RAD_ is substantially smaller than π_TRUE_ in both cases (π_RAD_ = 6.2 % and 3.5 %, respectively).

**Fig. 4:**
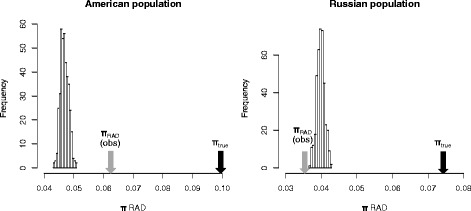
A comparison between simulations and empirical data in highly diverse populations. Distributions show the π_RAD_ values expected under the neutral panmictic model with θ = 9.7 % (*American population, on the left*) and θ = 7.4 % (*Russian population, on the right*). The black arrows indicate the true polymorphism values (π_true_) in the two populations. The grey arrows indicate the observed π_RAD_ values. Each distribution was computed from 400 simulations

To assess the pertinence of the neutral panmictic model to predict the bias under such high levels of polymorphism, we computed the distribution of π_RAD_ values expected with population mutation rates of 9.7 % and 7.4 % (corresponding to the true polymorphism values in the two populations). The results (Fig. 4) show that the model captures a large part of the bias (the expected π_RAD_ values are much closer to the observed π_RAD_ values than to the π_TRUE_ values), but not very accurately: in both populations, the observed π_RAD_ values fall out of their expected distribution. The discrepancy between the data and the model predictions is strongest in the American population, where the bias is less intense than predicted. In the Russian population, the bias is slightly more intense than predicted, although the observed value falls very close to the expected distribution.

### Partial corrections of the RAD-seq bias through ABC under a neutral panmictic model

We explored the possibility of using simulations from the neutral panmictic model to correct, at least partially, the RAD-seq estimates of polymorphism, through Approximate Bayesian Computation. The principle of this approach is to use the simulated relation between the true polymorphism and some summary statistics (e.g. proportion of shared RAD loci between specimens, proportion of homozygous loci) to infer corrected polymorphism values from the values of these statistics in empirical data. We developed such an ABC approach and first performed a cross-validation test, that is, tested our ability to correctly infer the input parameter values of simulations using the simulated data itself (here the simulated data is treated as an observation and is thus called “pseudo-observed”).

**Fig. 5:**
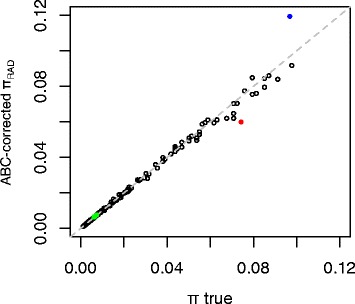
ABC corrections of the RAD-seq bias. The figure shows the relation between π_true_ and the corrected π_RAD_ values, that is, the θ parameter estimated by ABC. Black dots correspond to simulated data (cross-validation). Green dots represent *Drosophila melanogaster* populations. *Blue* and *red* dots represent American and Russian populations of *Schizophyllum commune*, respectively

Pseudo-observed data was generated with different θ values, and for each simulated data set, the observed RAD genetic distance was calculated, as well as the proportion of RAD loci shared between the two specimens. Using these two summary statistics, we were able to precisely infer the input parameter values (Fig. 5). Noteworthy, this cross validation is only a quality control, showing that the ABC approach can be used to retrieve the true polymorphism from the observed RAD data *if* the data follow the exact same model as the simulations. We then tested this approach on real data, using *in silico* RAD-seq from *D. melanogaster* and *S. commune* (Fig. 5). The ABC-corrected RAD polymorphism value was close to the true polymorphism in *D. melanogaster*, where the RAD-seq bias was very low anyway. In *S. commune*, ABC estimates for the two populations are also much closer to the true polymorphism values than the uncorrected RAD values, with some discrepancies, as expected from the above discussed deviations of these populations from the neutral panmictic model. While the uncorrected RAD polymorphism was about half of the true polymorphism, the ABC corrected values were only 20 % away from the true values: slightly too low in the American population, slightly too large in the Russian population.

## Discussion

Building on previous studies [8, 9], we further explored here the impact of allele drop-out on the estimation of genetic diversity from RAD data. We first confirmed earlier findings based on simulations in a neutral and panmictic model: RAD-based estimates of diversity are lower than the true polymorphism, and this bias becomes more pronounced as the true polymorphism increases. Using more elaborate models, we further showed that deviations from the neutral and panmictic model can have complex and contradictory outcomes. Assigning different degrees of polymorphism to different regions of the genome, which mimics the effects of natural selection on genomic variation, tends to exacerbate the RAD-seq bias. This probably results from an excessive contribution to the data of the least polymorphic genomic regions, subject to the most intense purifying selection. We also simulated sampling of specimens from more or less isolated subpopulations, and thus showed that population structure should mitigate the bias. In other words, RAD-seq data tends to under-estimate divergence *within* but not *between* populations.

We used “ideal” empirical data, that is, RAD-seq data obtained from *in silico* digestion of full genomes from natural populations, to assess potential deviations from the neutral and panmictic model. Data from the fruit fly *D. melanogaster* confirmed that the bias is of negligible importance when the polymorphism is low, offering little power to assess the validity of the model. On the contrary, in the fungi *S. commune*, where the true polymorphism approaches 10 %, the bias is severe, producing a 50 % underestimation of the diversity. The neutral and panmictic model captures most of this effect, but the observed RAD-based values nevertheless fall out of the model predictions.

We investigated whether these deviations might be due to selection or spatial structure. In the American population, where the bias was weaker than expected, one specimen (from Florida) was significantly differentiated from all others (from Michigan, Fig. 1 in [13]). However, excluding this specimen from the analysis only has a minor effect on the bias for this population (not shown), suggesting the discrepancy with our theoretical expectation is not necessarily explained by population structure. We also explored whether heterogeneity in θ along the genome might occur in this data set. We found that the distribution of the SNPs across RAD tags was indeed significantly more heterogeneous than expected under a Poisson process (Additional file 1: Figure S1). Moreover, distributions of RAD distances were always significantly more heterogeneous in the Russian population, which might contribute to the excessive bias observed in this population (Additional file 1: Figure S2).

The fact that simulations can capture the RAD-seq bias, at least in part, opens the possibility of correcting estimations through an ABC approach. We developed such an approach based on simulations from the neutral panmictic model, where the number of parameters to be estimated is low enough. The results are encouraging: the corrected RAD-polymorphism values are much closer to the real polymorphism than the raw values. However, in accordance with the above-discussed deviations from the model, the corrections are inaccurate. It is clear that robust estimations of diversity measures from RAD-seq data would require more elaborate ABC models, including the potential effects of population structure and selection, or other, yet unidentified, relevant parameters. However, our simulations suggest that a given observed RAD polymorphism might be indicative of a certain θ value if the population is panmictic, a smaller θ if individuals were sampled from slightly divergent populations, or a larger θ if selection produced strong heterogeneity in θ along the genome. In other words, an excessive number of parameters, with contradictory effects, might prevent convergence of the model toward a single optimal solution.

## Conclusion

Our analysis confirmed the tendency of RAD data to underestimate polymorphism. Regardless of the model used, simulations indicate this bias is of minor importance when the polymorphism is below 2 %, which is the case in most species, at least in animals [17]. *In silico* RAD experiments on full genome data from natural populations confirm this prediction, which would undoubtedly be reinforced by more realistic RAD datasets, where all sorts of additional biases, from technical issues at the bench to downstream bioinformatics, introduce more important sources of uncertainty [3–5, 18]. Nevertheless, when the polymorphism is large the RAD-seq bias becomes of significant concern, and needs to be kept in mind. While ABC-corrections based on a neutral and panmictic model can partially solve the problem, deviations from this model introduce some uncertainty in these corrections. Developing more robust corrections, although desirable, might face the difficulty of estimating too many parameters with insufficient data.

Once a bias has been found to affect a widely used technique such as RAD-seq, it seems crucial to understand its causes and evaluate its range, which was the purpose of the present study. This being said, one should also keep in mind that any set of molecular markers, from single genes to “random” shotgun sequencing, also present various kinds of bias, because it is virtually impossible to randomly sample genomic data. Until full genomes will be made achievable at reasonable costs for population genomics studies, RAD-seq thus remains, in our opinion, an optimal compromise.

## Additional file

Additional file 1: Table S1. Genomic sequences used for the *in silico* RAD-seq experiments. 2. Polymorphism heterogeneity along the genome of Schizophyllum commune. **Figure S1.** Theoretical and observed distributions of genetic distances (number of SNPs between RAD tags) between two American *S. commune* individuals (A10 and A13, on the left) and between 2 Russian individuals (K1 and K3, on the right). Blue: observed distribution of genetic distances; red: Poisson distribution, expected under a model of homogeneous polymorphism along the genome. Kolmogorov-Smirnov test, *D* = 0.2404, *p*-value < 2.2e-16 and *D* = 0.3881, *p*-value < 2.2e-16. **Figure S2.** Observed distributions of genetic distances (number of SNPs between RAD tags) between 2 American *S. commune* individuals (A10 and A13) and 2 Russian individuals (K1 and K3). Kolmogorov-Smirnov test, *D* = 0.2916, *p*-value < 2.2e-16. The figure shows that the distribution of RAD distances is more heterogeneous in the Russian population. 3. Examples of command lines for ms and seq-gen. (DOC 378 kb)

## Abbreviations

RAD-seq: Restriction Associated DNA sequencing
