# Supplementary material for: How and how much does RAD-seq bias genetic diversity estimates?
Source: BMC Evol Biol. 2016 Nov 8;16:240. doi: 10.1186/s12862-016-0791-0 (PMC5100275; doi:10.1186/s12862-016-0791-0)
Supplement: Additional file 1: Table S1. — Genomic sequences used for the in silico RAD-seq experiments. 2. Polymorphism heterogeneity along the genome of Schizophyllum commune. Figure S1. Theoretical and observed distributions of genetic distances (number of SNPs between RAD tags) between two American S. commune individuals (A10 and A13, on the left) and between 2 Russian individuals (K1 and K3, on the right). Blue: observed distribution of genetic distances; red: Poisson distribution, expected under a model of homogeneous polymorphism along the genome. Kolmogorov-Smirnov test, D = 0.2404, p-value < 2.2e-16 and D = 0.3881, p-value < 2.2e-16. Figure S2. Observed distributions of genetic distances (number of SNPs between RAD tags) between 2 American S. commune individuals (A10 and A13) and 2 Russian individuals (K1 and K3). Kolmogorov-Smirnov test, D = 0.2916, p-value < 2.2e-16. The figure shows that the distribution of RAD distances is more heterogeneous in the Russian population. 3. Examples of command lines for ms and seq-gen. (DOC 378 kb) [file 12862_2016_791_MOESM1_ESM.doc]

**Supporting information**

**How and how much does RADseq bias genetic diversity estimates?**

Marie Cariou^1,2^, Laurent Duret^1^ & Sylvain Charlat^1^.

^1^ Univ Lyon ; Université Claude Bernard Lyon 1 ; CNRS ; UMR 5558, Laboratoire de Biométrie et Biologie Evolutive, 43 boulevard du 11 novembre 1918, Villeurbanne F-69100, France

^2^ Current address: University of Namur, Department of Biology, Rue de Bruxelles 61, 5000 Namur, Belgium

*Corresponding author. email: marie.cariou@unamur.be, telephone: +32 81/72 43 59

| **Populations** | **Individuals** | **Reφes** |
| --- | --- | --- |
| ***Drosophila melanogaster*** | |  |
| ZI | ZI91, ZI261, ZI268, ZI468 | (Poo*l et a*l. 2012) |
| GU | GU2, GU6, GU7, GU10 |  |
| KR | KR4N, KR7, KR42, KR39 |  |
| RG | RG2, RG10, RG15, RG19 |  |
| ***Schizophillum commune*** | |  |
| american | A10, A13, A3, A4, A7, A8, B1, B3, B5, B6, Mi1, Fl | (Baranov*a et a*l. 2015) |
| russian | K1, K3, K4, M1, M2, M3, M4, M5, M6, Mos, S1, S4 |  |

**Table S 1.** Genomic sequences used for the *in silico* RAD-seq experiments.

**Polymorphism heterogeneity along the genome of *Schizophyllum commune***

To better understand to what extend selection might affect the RAD-seq bias in populations of *S. commune*, we assessed the heterogeneity in polymorphism along the genome. To do so, we measured the number of SNPs at all RAD loci and computed their distribution for all pairs of haploid individuals from the same population. We compared these distributions with theoretical Poisson distributions using a Kolmogorov-Smirnov test. This showed that polymorphism was significantly more heterogeneous than predicted by a Poisson distribution in *S. commune* genomes, both in American and Russian populations, as expected given that these are real genomes on which selection has acted. This result is illustrated in Figure S1 for 2 pairs of individuals chosen randomly within each population (A10 and A13, and K1 and K3). Similar results were obtained for 4 more pairs (not shown).

Distributions of the genetic distances across loci were also compared between American and Russian populations. Kolmogorov-Smirnov tests show that these distributions are always significantly more heterogeneous in the Russian population, for the 25 comparisons computed (Figure S2 illustrates the comparison of pair A10-A13 with pair K1-K3). In contrast, comparisons between distributions based on pairs of individuals chosen within the same populations were often not significantly different (not shown). Thus, the polymorphism was generally more heterogeneous along the genome of specimens from the Russian populations.

Figure S1. Distributions of genetic distances (number of SNPs between RAD tags) among two American *S. commune* individuals (A10 and A13) and 2 Russian individuals (K1 and K3). Blue: observed distribution of genetic distances; red: Poisson distribution, expected under a model of homogeneous polymorphism along the genome. Kolmogorov-Smirnov test, D = 0.2404, p-value < 2.2e-16 and D = 0.3881, p-value < 2.2e-16.

Figure S 2. Distributions of genetic distances (number of SNPs between RAD) between 2 American *S. commune* individuals (A10 and A13) and 2 Russian individuals (K1 and K3). Kolmogorov-Smirnov test, D = 0.2916, p-value < 2.2e-16.

### Examples of command lines for *ms* and *seq-gen*:

ms 4 1000 -t *θ* -T -I 2 2 2 (-n 2 *1* -n 1 *1* -ej *t* 2 1)>ms_output

tail -n +4 ms_output | grep -v // > treefile

seq-gen -mHKY -l 10000 -f 0.25 0.25 0.25 0.25 -t 1 -s *θ *<treefile

### References

Baranova MA, Logacheva MD, Penin AA *et al.* (2015) Extraordinary Genetic Diversity in a Wood Decay Mushroom. , **32**, 2775–2783.

Csilléry K, François O, Blum MGB (2012) Abc: An R package for approximate Bayesian computation (ABC). *Methods in Ecology and Evolution*, **3**, 475–479.

Hudson RR (2002) Generating samples under a Wright-Fisher neutral model of genetic variation. *Bioinformatics (Oxford, England)*, **18**, 337–338.

Pool JE, Corbett-Detig RB, Sugino RP *et al.* (2012) Population Genomics of Sub-Saharan Drosophila melanogaster: African Diversity and Non-African Admixture. *PLoS Genetics*, **8**.

Rambaut A, Grassly NC (1997) Seq-Gen: an application for the Monte Carlo simulation of DNA sequence evolution along phylogenetic trees. *CABIOS*, **13**, 235–238.
